# Integrative taxonomy reveals two new species of whiptail catfishes *Loricaria* (Siluriformes: Loricariidae) from northeastern Brazil

**DOI:** 10.1111/jfb.70395

**Published:** 2026-04-08

**Authors:** Ananda. C. Serejo‐Saraiva, Nivaldo M. Piorski, Felipe P. Ottoni, Mark H. Sabaj, Sergio M. Q. Lima

**Affiliations:** ^1^ Programa de Pós‐Graduação em Sistemática e Evolução, Universidade Federal do Rio Grande do Norte Natal Brazil; ^2^ Laboratório de Ecologia e Sistemática de Peixes, Departamento de Biologia Universidade Federal do Maranhão São Luís Maranhão Brazil; ^3^ Laboratório de Sistemática e Ecologia de Organismos Aquáticos, Campus Chapadinha Universidade Federal do Maranhão Chapadinha Maranhão Brazil; ^4^ NRF‐South African Institute for Aquatic Biodiversity (NRF‐SAIAB) Makhanda South Africa; ^5^ Department of Ichthyology The Academy of Natural Sciences of Drexel University Philadelphia Pennsylvania USA; ^6^ Laboratório de Ictiologia Sistemática e Evolutiva, Departamento de Botânica e Zoologia Universidade Federal do Rio Grande do Norte, Lagoa Nova Natal Brazil

**Keywords:** Cerrado fishes, Itapecuru River, Loricariinae, Maranhão State, Munim River, taxonomy

## Abstract

Two new species of whiptail catfish, *Loricaria* (Siluriformes: Loricariidae), were discovered in the Munim and Itapecuru river basins, Maranhão State, northeastern Brazil, through an integrative taxonomic approach combining morphology and mitochondrial DNA. Linear discriminant analysis revealed three morphometrically distinct groups, and *coxI*‐based phylogeny identified four genetically divergent lineages of *Loricaria* in northeastern Brazil. The new species are clearly distinguished from their closest congeners, *L. turi* and *L. parnahybae*, by unique diagnostic traits, including abdominal plate patterns, pectoral girdle coverage and the shape of the postorbital notch. Genetic divergences among lineages, estimated as mean interspecific K2P distance, ranged from 2.2% to 4.1%, similar thresholds commonly applied for species delimitation in Loricariidae. Both species exhibit restricted distributions and are endemic to river basins under increasing anthropogenic pressure. These results underscore the underestimated diversity of *Loricaria* in northeastern Brazil and reinforce the role of coastal drainages as areas of endemism and speciation.

## 1 INTRODUCTION

The subfamily Loricariinae is a highly diverse group of Neotropical fishes currently comprising 45 genera and approximately 230 species (van der Laan & Fricke, 2025), commonly referred to as whiptail catfish or ‘cascudo‐viola’ in Brazil. The genus *Loricaria* Linnaeus, 1758 is one of the most taxonomically challenging, with species often displaying subtle external morphological variation and overlapping morphometric characters (Isbrücker, 1981; Londoño‐Burbano et al., 2023; Saraiva et al., 2021; Thomas, 2011).

Isbrücker (1981) diagnosed *Loricaria* by the presence of filamentous papillae on the upper and lower lips, and three to four premaxillary teeth, twice the size of dentary teeth; later studies reported up to six premaxillary teeth per ramus excluding gaps (e.g., Thomas, 2011; Provenzano & Sabaj, 2024). *Loricaria* currently comprises 20 valid species (Table 1) distributed across the major South American river systems, with several recent additions described from the Amazon, Tocantins, Orinoco, Paraná, and northeastern Brazilian basins. Isbrücker (1981) conducted the first taxonomic revision of the genus and Thomas (2011) performed a comprehensive review and morphological phylogenetic analysis in an unpublished doctoral thesis.

**TABLE 1:** Valid species of *Loricaria* (Fricke et al., 2025).

Species
*Loricaria apeltogaster* Boulenger, 1895
*Loricaria birindellii* Thomas & Sabaj, 2010
*Loricaria cataphracta* Linnaeus, 1758
*Loricaria clavipinna* Fowler, 1940
*Loricaria coximensis* Rodriguez, Cavallaro & Thomas, 2012
*Loricaria cuffyi* Londoño‐Burbano, Urbano‐Bonilla & Thomas, 2020
*Loricaria holmbergi* Rodriguez & Miquelarena, 2005
*Loricaria lata* Eigenmann & Eigenmann, 1889
*Loricaria luciae* Thomas, Rodriguez, Carvallaro, Froehlich & Castro, 2013
*Loricaria lundbergi* Thomas & Rapp Py‐Daniel, 2008
*Loricaria nickeriensis* Isbrücker, 1979
*Loricaria nimairaco* Londoño‐Burbano, Urbano‐Bonilla, Thomas & Britto, 2023
*Loricaria parnahybae* Steindachner, 1907
*Loricaria piracicabae* Ihering, 1907
*Loricaria pumila* Thomas & Rapp Py‐Daniel, 2008
*Loricaria simílima* Regan, 1904
*Loricaria spinulifera* Thomas & Rapp Py‐Daniel, 2008
*Loricaria thomasi* Provenzano & Sabaj, 2024
*Loricaria tucumanensis* Isbrücker, 1979
*Loricaria turi* Saraiva, Abreu, Ottoni & Piorski, 2021

*Loricaria* is widely distributed across the Amazon, Orinoco, and Paraná‐Paraguay basins, and in the smaller coastal drainages of the Guiana and Brazilian Shields (Isbrücker, 1981; Londoño‐Burbano et al., 2023; Thomas et al., 2013). Partly due to its broad distribution, the taxonomy of *Loricaria* remains unresolved, especially in under‐sampled areas such as the coastal basins of northeastern Brazil.

The river basins of northeastern Brazil form a complex network influenced by the Amazon, Cerrado, and Caatinga biomes. Historically under‐sampled and under‐studied, their ichthyofauna is still being discovered (Abreu et al., 2019; Rosa et al., 2003; Zawadzki et al., 2017). Currently, only two valid species of *Loricaria* are recognized in this region: *L. turi*, endemic to the Turiaçu River basin, and *L. parnahybae*, endemic to the Parnaíba River basin (Saraiva et al., 2021). However, Saraiva et al. (2021) have identified morphologically distinct forms in adjacent basins.

Species delimitation in *Loricaria* has been hindered by the low variability of external morphology, with characters often overlapping between species or displaying high intraspecific variation (Saraiva et al., 2021). In this context, integrative approaches combining morphological and molecular data have proven essential for revealing hidden diversity and clarifying species boundaries in loricariids (Castellanos‐Mejía et al., 2024; Fisch‐Muller et al., 2018; Lustosa‐Costa et al., 2024).

In this study, we applied an integrative taxonomic approach to describe two new species of *Loricaria* from the Itapecuru and Munim River basins in northeastern Brazil. We present morphological and molecular evidence supporting the taxonomic validity of these species and contributing to the knowledge of *Loricaria* diversity in the region.

## 2 MATERIALS AND METHODS

### 2.1 Ethics statement

This study was based exclusively on specimens already deposited in ichthyological collections, with no collection of live specimens or addition of material to the collections, therefore approval by an institutional Animal Ethics Committee (CEUA) was not required.

### 2.2 Morphometrics analyses

Morphometric measurements were obtained using a digital calliper with 0.1 mm precision and are presented as percentages of standard length (SL) or head length (HL). Morphometric measurements and meristic counts followed Thomas & Rapp Py‐Daniel (2008). Terminology and counts of dermal plates followed Thomas & Perez (2010). All morphometric and meristic data used for interspecific comparisons were obtained by the authors from examined museum specimens listed in the Comparative material. When direct examination was not possible, comparative information was obtained from original species descriptions and relevant taxonomic literature. Colour descriptions were based on specimens preserved in ethanol. Institutional abbreviations followed Sabaj (2025).

A linear discriminant analysis (LDA) was performed to evaluate whether morphological variation is sufficient to support separation among *Loricaria* species from northeastern Brazil. To remove the allometric effect from morphometric data, Burnaby's method (Burnaby, 1966) was applied, which projects the data onto a plane orthogonal to the size vector. The LDA was conducted in the R statistical environment (R Core Team, 2024) using the packages MASS (Venables & Ripley, 2002) and ggplot2 (Wickham, 2016).

### 2.3 Molecular analyses

Fin tissue samples deposited in the ichthyological tissue collection of the Universidade Federal do Rio Grande do Norte (TIUFRN; Table 2) were used. DNA extraction followed the modified saline protocol of Bruford et al. (1992). Fragments of the mitochondrial cytochrome c oxidase subunit I gene (*cox1*) were amplified using the primers FISH‐F1 and FISH‐R1 (Ward et al., 2005). PCR reactions were performed in a total volume of 25 μL, consisting of 14.5 μL of 2 × Master Mix Green GoTaq (Promega), 1.0 μL of each primer, 6.5 μL of ultrapure water, and 2 μL of DNA. The amplification protocol included an initial denaturation at 94°C for 4 min, followed by 40 cycles of: denaturation at 95°C for 30 s, annealing at 50°C for 20 s, and extension at 72°C for 90 s, with a final extension step at 72°C for 10 min. PCR products were visualized on 1% agarose gels, and successful amplifications were purified and sequenced bidirectionally by Macrogen Inc. (http://www.macrogen.com).

**TABLE 2:** Voucher specimens and novel tissue samples generated in this study of *Loricaria*, all from Brazil.

Species	Voucher	Tissue number	Locality	Coordinates	Genbank
*Loricaria* sp. ‘Munim’	CICCAA 07589	TIUFRN 6007	Rio Munim, Chapadinha, MA	03°42′20.4″S 43°31′46.3″W	PX243302
*Loricaria* sp. ‘Munim’	CICCAA 07590	TIUFRN 6008	Rio Munim, Chapadinha, MA	03°42′20.4″S 43°31′46.3″W	PX243301
*Loricaria* sp. ‘Itapecuru’	CPUFMA 4160	TIUFRN 6172	Rio Itapecuru, Rosário, MA	02°59′48.3″S 44°14′24.7″W	PX243298
*Loricaria* sp. ‘Itapecuru’	CPUFMA 4160	TIUFRN 6173	Rio Itapecuru, Rosário, MA	02°59′48.3″S 44°14′24.7″W	PX243297
*Loricaria* sp. ‘Itapecuru’	CPUFMA 4160	TIUFRN 6174	Rio Itapecuru, Rosário, MA	02°59′48.3″S 44°14′24.7″W	PX243296
*Loricaria* sp. ‘Itapecuru’	CPUFMA 4160	TIUFRN 6169	Rio Itapecuru, Rosário, MA	02°59′48.3″S 44°14′24.7″W	PX243295
*L. turi*	CFPE 1286	TIUFRN 6298	Rio Turiaçu, Santa Helena, MA	02°13′39″S 45°18′03″W	PX243300
*L. turi*	CFPE 1335	TIUFRN 6300	Rio Turiaçu, Santa Helena, MA	02°13′39″S 45°18′03″W	PX861347
*L. turi*	CFPE 1336	TIUFRN 6303	Rio Turiaçu, Santa Helena, MA	02°12′03″S 45 W 17′ 01″	PX243299
*L. parnahybae*	UFRN 1841	TIUFRN 1958	Rio Sambito, Aroazes, PI	06°11′32.1″S 41°59′38.3″W	PX243309
*L. parnahybae*	UFRN 1841	TIUFRN 1959	Rio Sambito, Aroazes, PI	06°11′32.1″S 41°59′38.3″W	PX243308
*L. parnahybae*	UFRN 5195	TIUFRN 4576	Rio Itaim, Aroeiras, PI	07°17′39.4″S 41°33′51.4″W	PX243304
*L. parnahybae*	UFRN 5195	TIUFRN 4575	Rio Itaim, Aroeiras, PI	07°17′39.4″S 41°33′51.4″W	PX243305
*L. parnahybae*	N/A	TIUFRN 4858	Riacho do Muquem, Barão do Grajaú, MA	06°41′10.3″S 43°16′37.2″W	PX243303
*L. parnahybae*	UFRN 1841	TIUFRN 1960	Rio Sambito, Aroazes, PI	06°11′32.1″S 41°59′38.3″W	PX243307
*L. parnahybae*	UFRN 5195	TIUFRN 4574	Rio Itaim, Aroeiras, PI	07°17′39.4″S 41°33′51.4″W	PX243306

*Note*: The Locality column indicates river basin, municipality and state (MA, Maranhão; PI, Piauí). Tissue samples are deposited in the Coleção de Tecidos Ictiológicos da Universidade Federal do Rio Grande do Norte (TIUFRN). Voucher specimens are deposited in CPUFMA (Coleção de Peixes da Universidade Federal do Maranhão), CICCAA (Coleção Ictiológica do Centro de Ciências Agrárias e Ambientais da Universidade Federal do Maranhão), and CFPE (Coleção de Tecidos e DNA da Fauna Maranhense, Universidade Estadual do Maranhão). N/A, not applicable.

Consensus sequences were generated by assembling forward and reverse reads using SeqMan (DNASTAR Lasergene). Sequences were then manually edited to correct ambiguous or erroneous base calls, eliminate misalignments, and trim low‐quality regions at sequence ends. As a result of quality trimming, the final alignment comprised 579 base pairs (bp).

Alignments were performed using the ClustalW algorithm in MEGA X (Kumar et al., 2018) with default parameters. Genetic distances were calculated in MEGA X using the Kimura 2‐parameter model (Kimura, 1980). Additional sequences were obtained from GenBank (Table S1), and voucher specimen information for these sequences follows the original database records. Sequences of *Pseudohemiodon laticeps* (Regan, 1904) (GenBank KU288807.1 and KU288806.1) were used as an outgroup, following Covain et al. (2016).

### 2.4 Species delimitation analyses

Four species delimitation methods were applied, including two distance‐based and two coalescent‐based approaches, respectively: ABGD (Automatic Barcode Gap Discovery; Puillandre et al., 2012), ASAP (Assemble Species by Automatic Partitioning; Puillandre et al., 2021), bPTP (Bayesian implementation of the Poisson Tree Process; Zhang et al., 2013), and GMYC single‐threshold (General Mixed Yule Coalescent; Fujisawa & Barraclough, 2013).

ABGD and ASAP (distance‐based) analyses used genetic distance matrices as input. The ABGD analysis was conducted using the online server at https://bioinfo.mnhn.fr/abi/public/abgd/abgdweb.html, employing the Kimura two‐parameter model (Kimura, 1980). The ASAP analysis was also performed online at https://bioinfo.mnhn.fr/abi/public/asap/asapweb.html, using the same substitution model under default algorithm settings.

The bPTP and GMYC (coalescent‐based) analyses used phylogenetic trees as input. For bPTP, a maximum likelihood tree generated in IQ‐Tree (Trifinopoulos et al., 2016) based on a dataset of unique haplotypes was submitted to the online server at http://species.h-its.org/ptp/. For GMYC, an ultrametric tree was inferred using BEAST v2.7.5 (Bouckaert et al., 2019), under the T92+G substitution model (Tamura, 1992; Yang, 1994) selected by the Bayesian Information Criterion (BIC), a strict molecular clock with a fixed evolutionary rate of 1, and the Yule process as the speciation prior. All other parameters were kept at default values. Markov Chain Monte Carlo (MCMC) chains were run for 50 million generations, sampling every 5,000 generations. Convergence (ESS > 200) was assessed using Tracer v1.7.1 (Raumbaut et al., 2018). The consensus tree was obtained in TreeAnnotator v2.6.7 (Drummond & Rambaut, 2007), with 25% burn‐in, and visualized in FigTree v1.4.4. (Rambaut, 2018) GMYC analysis was conducted in R using the *splits* package (Ezard et al., 2009). A lineage was considered distinct when at least three of the delimitation methods used yielded concordant results.

### 2.5 Conservation assessment

The conservation status of the species was assessed following the IUCN Red List Categories and Criteria (IUCN, 2024). The extent of occurrence (EOO) was calculated based on georeferenced locality records using the website https://geocat.iucnredlist.org/. The resulting values were subsequently interpreted according to the IUCN guidelines.

## 3 RESULTS

### 3.1 Morphometrics analysis

**FIGURE 1:**
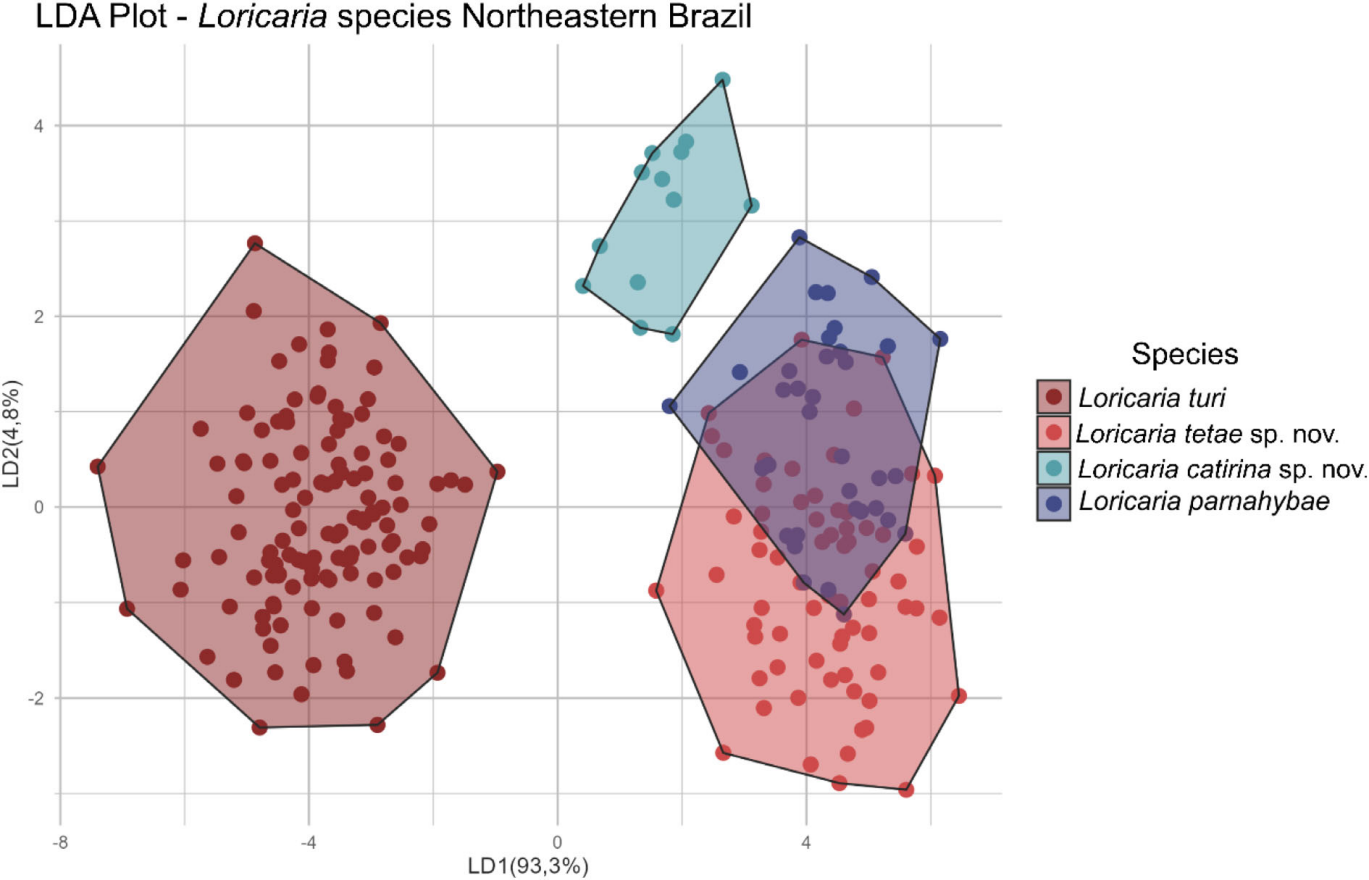
Linear discriminant analysis (LDA) of *Loricaria* specimens from northeastern Brazil. Legend shows groups arranged geographically from west (*L. turi*) to east (*L. parnahybae*).

The morphometric analysis included 239 individuals of *Loricaria* from northeastern Brazil, comprising 103 specimens of *L. turi* (Turiaçu basin), 36 of *L. parnahybae* (Parnaíba basin), 68 of *Loricaria teteae* sp. nov. (Itapecuru basin), and 13 of *Loricaria catirina* sp. nov. (Munim basin). Linear discriminant analysis (LDA) revealed complete separation among three morphologically distinct groups (Figure 1): the first composed of *L. turi* specimens, the second of *L. catirina* sp. nov. and the third comprising *L. parnahybae* and *L. teteae* sp. nov. The first LDA axis accounted for 93.3% of the total variation and was mainly influenced positively by post‐anal distance and basicaudal plate length, and negatively by maximum and minimum orbital diameters, whereas the second axis explained 4.85% of the variation and was primarily influenced by post‐dorsal distance and body depth.

### 3.2 Molecular analysis

**FIGURE 2:**
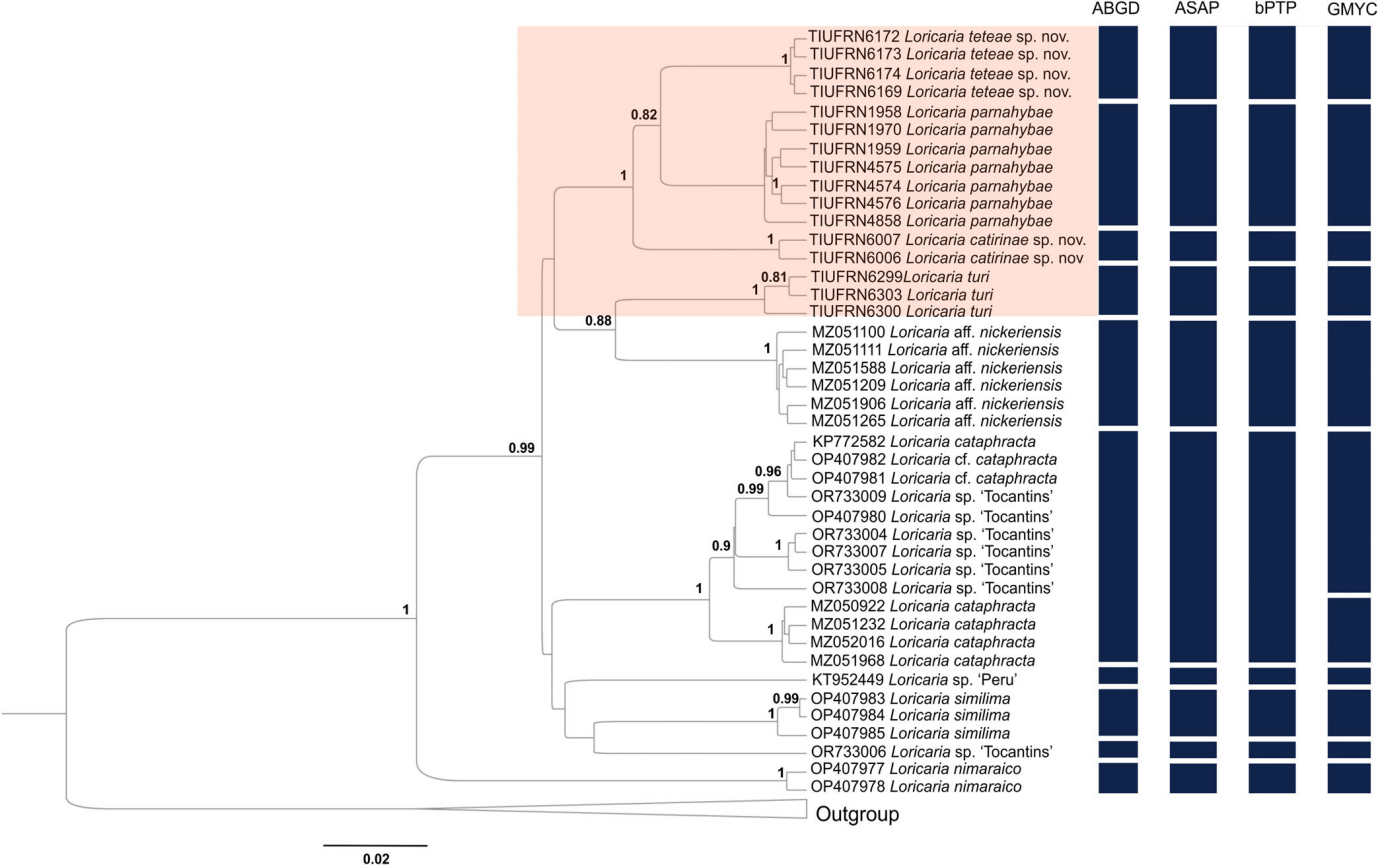
Bayesian inference tree based on a 579 bp fragment of the mitochondrial *cox1* marker for species of the genus *Loricaria*, illustrating the results of four species delimitation methods (ABGD, ASAP, bPTP and GMYC). Posterior probability values are shown at the nodes. The orange shaded area highlights the northeastern lineages.

In the Bayesian tree inferred from a 579 bp fragment of the *cox1* gene (42 individuals), three major clades were identified (Figure 2). Specimens from northeastern Brazil occur exclusively within one of these major clades, but do not form a monophyletic group, as they are distributed between two non‐sister lineages.

One of these lineages corresponds to the *Loricaria parnahybae* clade, which comprises *L. parnahybae*, *L. catirina,* and *L. teteae*. The second lineage corresponds to the *Loricaria nickeriensis* clade, including *L. turi* and *L*. aff. *nickeriensis*, a species from the Guiana Shield drainages. This result is consistent with the patterns observed in the LDA (Figure 1).

Species delimitation analyses (ABGD, ASAP, bPTP, and GMYC) further corroborated these relationships by recognizing four independent evolutionary units in the northeast (Figure 2). All methods consistently recovered *L. catirina* and *L. teteae* as distinct lineages, in addition to recognizing *L. turi* and *L. parnahybae* as separate lineages, congruent with their status as valid species.

Genetic distances among the northeastern lineages (Table 3) ranged from 2.2% to 4.1%. The highest divergence was observed between *Loricaria turi* and both *L. teteae* and *L. catirina* (4.1%), whereas the lowest divergence occurred between *L. catirina* and *L. parnahybae*, as well as between *L. teteae* and *L. parnahybae* (2.2%).

**TABLE 3:** Mean Kimura two‐parameter (K2P) genetic distances within and among *Loricaria* species from coastal river basins in northeastern Brazil based on *cox1* sequences.

Species	1	2	3	4
*Loricaria catirina* sp. nov.	**0.0**			
*Loricaria teteae* sp. nov.	0.03	**0.0**		
*Loricaria turi*	0.041	0.041	**0.0**	
*Loricaria parnahybae*	0.022	0.022	0.031	**0.0**

*Note*: Intraspecific distances (diagonal) are shown in bold.

*Loricaria catirina* shows genetic distances of 3.0% from *L. teteae*, 4.1% from *L. turi* and 2.2% from *L. parnahybae*. Likewise, *L. teteae* differs from *L. turi* by 4.1% and from *L. parnahybae* by 2.2%.

In addition to the *L. parnahybae* clade, which is closely related to the clade composed of *L. turi* and *L*. aff. *nickeriensis*, the phylogeny recovered two other major groups (Figure 2). The first is *L. nimaraico* (upper Amazon), the most basal species in this phylogeny. The second clade comprises specimens of *L. cataphracta* from different localities, which form a single lineage, along with a *Loricaria* lineage from the Tocantins River and the species *L. simillima*.

### 3.3 Morphometrics and molecular divergence analyses

Morphometric (LDA) and molecular analyses identified two distinct lineages associated with the Munim and Itapecuru river basins, respectively. In the LDA, *Loricaria teteae* (Itapecuru) partially overlaps with other lineages, whereas *Loricaria catirina* (Munim) occupies a distinct region of the morphospace. Molecular analyses recovered both lineages as reciprocally monophyletic. Species delimitation methods consistently recovered the Munim and Itapecuru lineages as separate operational taxonomic units.

### 3.4 Taxonomy

**ZooBank registration**. This work and the new species names herein are registered in ZooBank under the LSID: urn:lsid:zoobank.org:pub:363E1F46‐4FAC‐49D3‐9CD6‐E969DE3FD47C.

#### 3.4.1 *Loricaria catirina* sp. nov.

**FIGURE 3:**
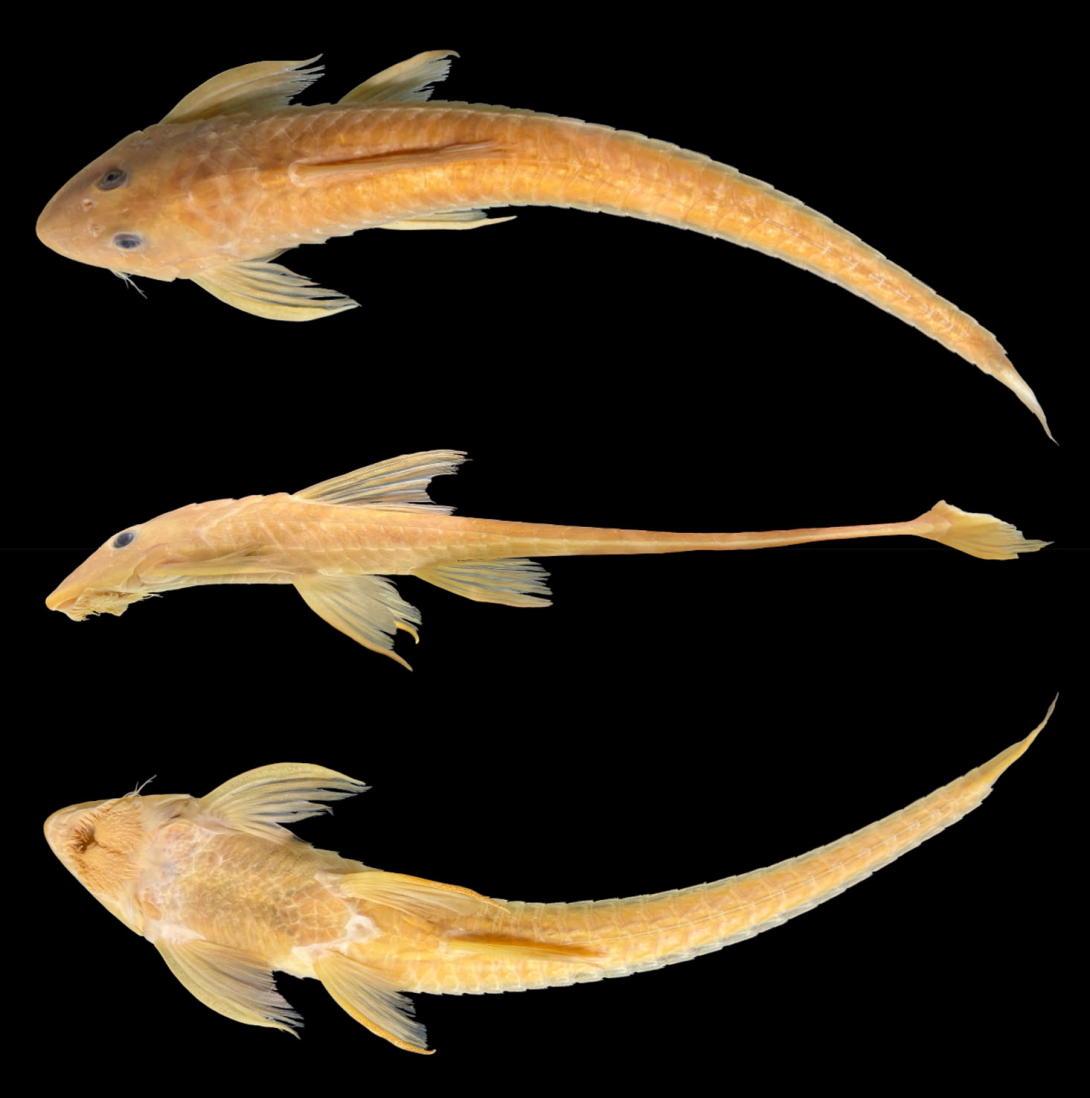
Holotype of *Loricaria catirina* MNRJ 56152, 181.31 mm standard length, male.

**FIGURE 4:**
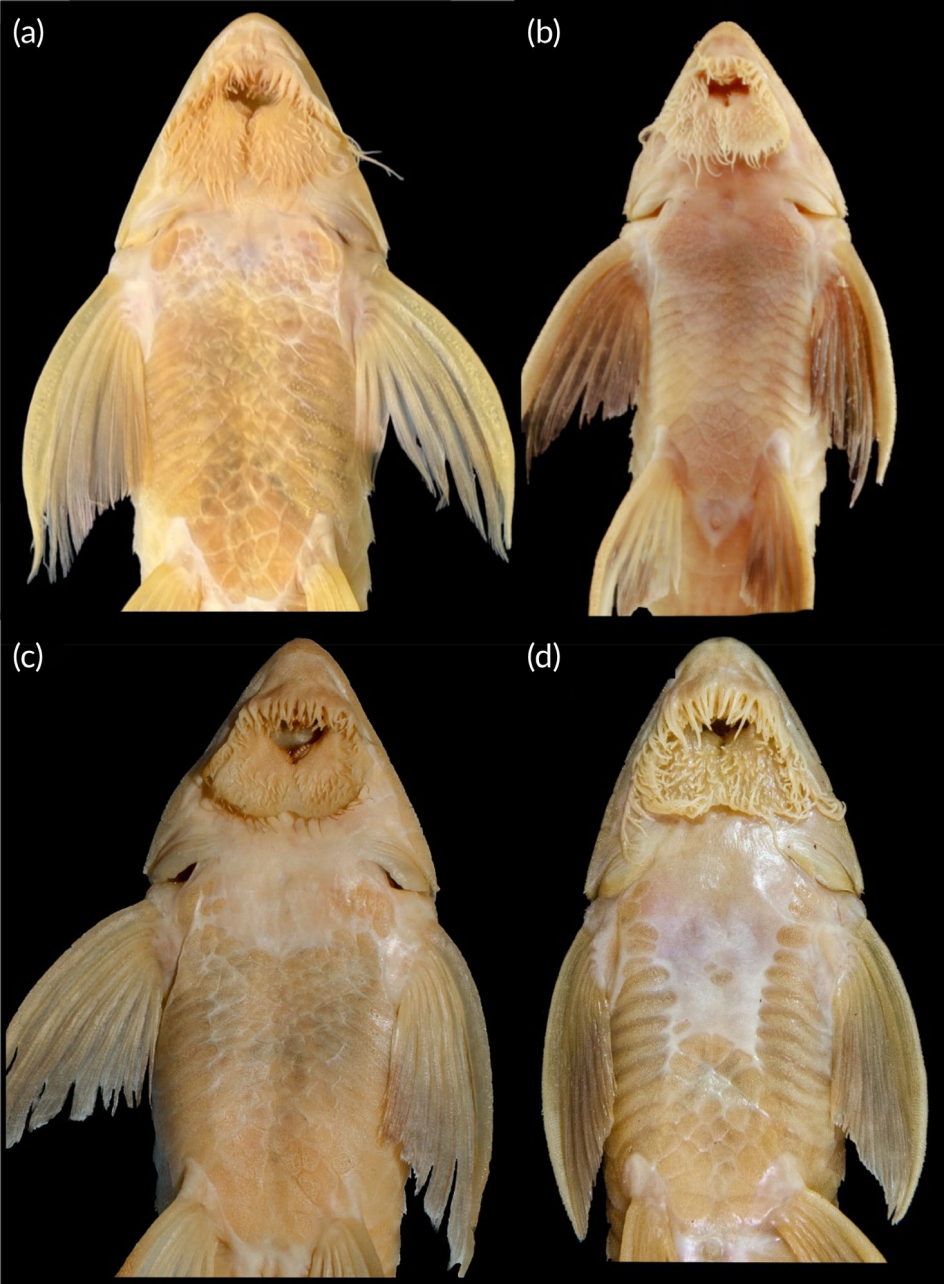
Variation in ventral abdominal plate morphology among *Loricaria* species from northeastern Brazil: (a) *L. catirina* sp. nov., (b) *L. teteae* sp. nov., (c) *L. turi,* and (d) *L. parnahybae*.

**FIGURE 5:**
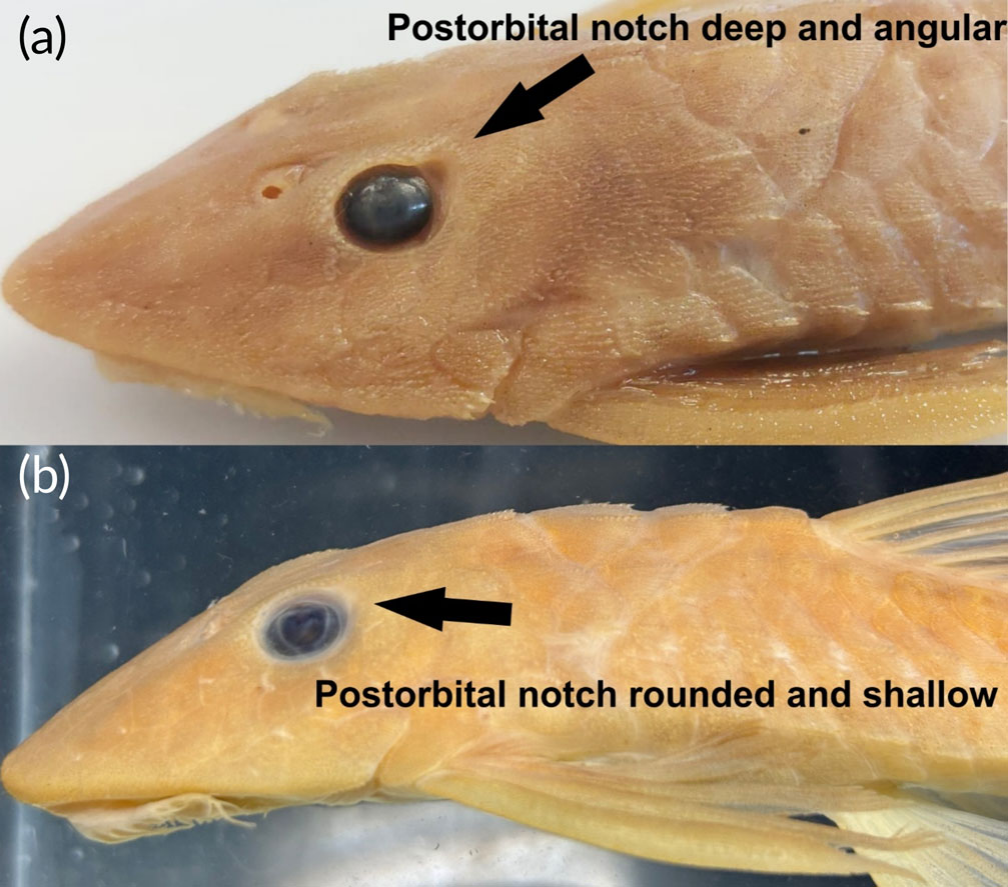
Lateral view of the head showing postorbital notch. (a) *Loricaria teteae* sp. nov. CPUFMA 822, post‐orbital notch deep and angular. (b) *Loricaria catirina* sp. nov. CPUFMA 1608, post‐orbital notch shallow and rounded.

**FIGURE 6:**
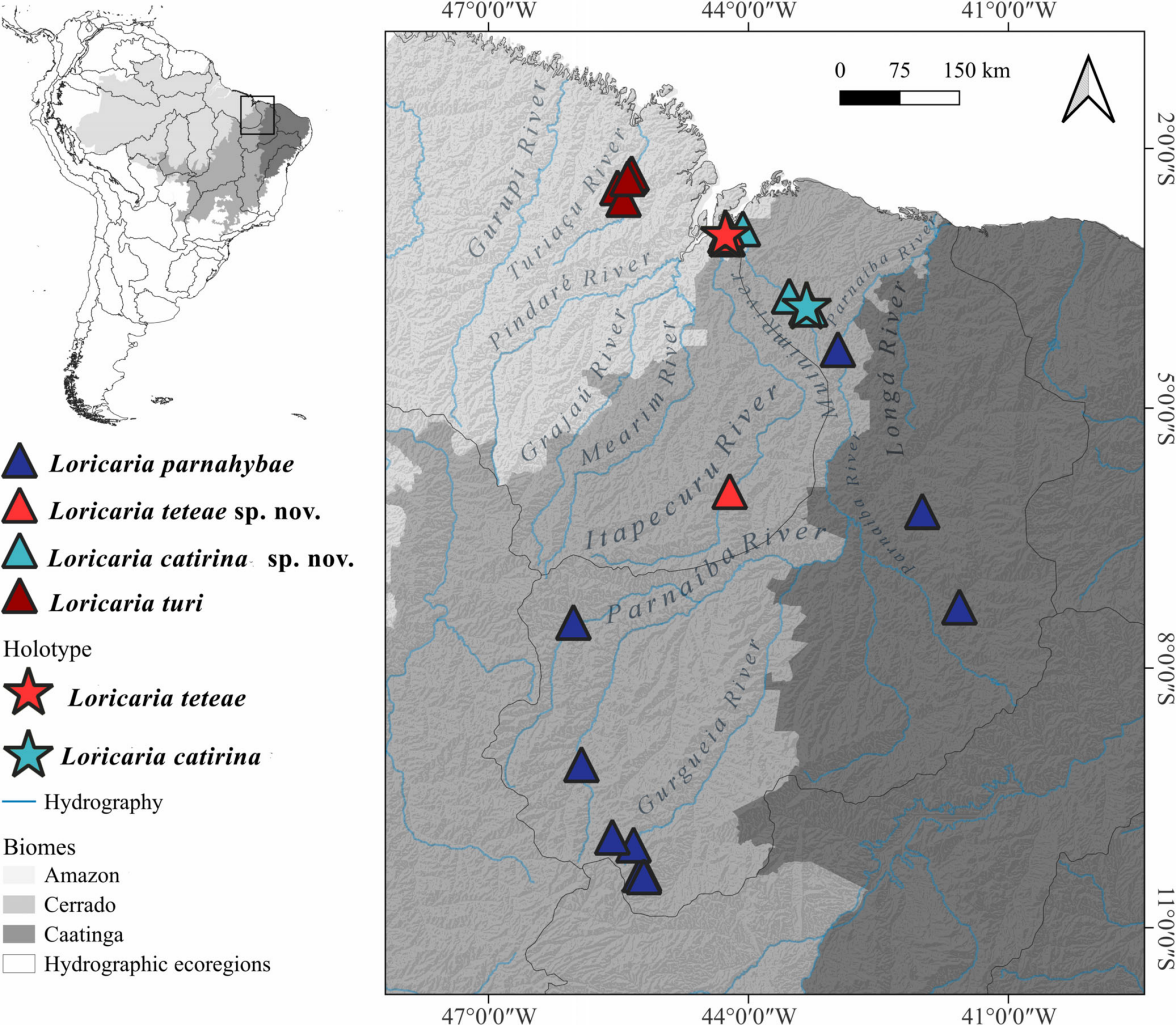
Distribution map of *Loricaria* species in northeastern Brazil.

Figures 3, 4, 5b, 6, Tables 4 and 5.

**TABLE 4:** Morphometric data for *Loricaria catirina* sp. nov. and *L. teteae* sp. nov. Values are presented as percentages of standard length (SL) or head length (HL).

	*Loricaria catirina* sp. nov.						*Loricaria teteae* sp. nov.					
	*N*	Holotype	Range		Mean	SD	*n*	Holotype	Range		Mean	SD
			Min	Max					Min	Max		
Standard length (mm)	18	181.3	153.7	202.9	176.8	16.9	68	188.1	123.9	249.8	188.1	35.8
		% SL						% SL				
Head length	18	19.9	18.9	22.1	19.8	0.7	68	19.1	16.1	21.4	19.2	0.9
Predorsal distance	18	28.7	27.3	31.9	28.6	1.0	68	28.1	26.1	31.0	27.8	1.0
Body width	18	8.2	8.01	10.1	8.7	0.5	68	9.3	7.8	11.1	9.5	0.8
Postdorsal length	18	57.5	55.6	63.0	57.3	1.6	68	62.7	54.8	63.9	58.6	1.6
Postanal length	18	48.5	46.7	54.5	48.8	2.0	68	53.6	46.6	56.7	50.5	1.6
Head width	18	14.5	13.4	15.8	14.3	0.7	68	14.4	12.2	17.1	13.9	0.7
Body width at post‐cleithral	18	13.6	11.5	13.8	13.0	0.7	68	13.5	11.0	15.8	12.7	0.8
Body width at dorsal spine origin	18	12.5	11.3	13.6	12.4	0.6	68	12.2	9.6	14.5	12.4	1.0
Body width at anal spine origin	18	10.7	9.2	10.3	11.0	0.5	68	10.7	8.4	12.4	9.9	0.7
Abdominal length	18	11.3	10.9	13.5	12.5	0.6	68	12.5	10.7	13.8	12.2	0.7
Thoracic length	18	14.4	12.6	16.1	14.5	0.9	68	12.1	11.0	16.8	13.9	1.0
Dorsal spine length	14	19.8	16.8	24.7	19.4	2.0	68	22.4	14.4	25.6	21.1	1.8
Pectoral spine length	14	17.3	15.3	18.6	16.7	0.9	68	17.8	15.4	19.5	17.6	0.7
Pelvic spine length	14	16.6	15.5	19.8	16.7	1.1	68	15.9	14.4	20.7	17.7	1.1
Anal spine length	17	15.2	11.6	19.4	16.7	1.1	68	17.2	11.1	23.3	17.3	1.5
	% HL							% HL				
Snout length	18	54.4	51.8	56.9	55.0	1.0	68	54.5	48.7	58.8	55.5	1.5
Minimum orbital diameter	18	17.3	16.2	18.9	17.6	0.7	68	15.1	12.6	18.5	15.3	1.5
Maximum orbital diameter	18	22.1	20.4	22.8	21.9	0.7	68	20.0	15.7	23.1	19.2	1.8
Head depth	18	40.2	37.5	42.4	39.3	1.5	68	42.8	38.6	53.1	42.3	2.7
Caudal peduncle depth	18	5.7	4.6	6.3	5.7	0.4	68	7.4	5.5	8.2	6.6	0.6
Internares distance	18	8.4	7.05	11.5	9.8	1.1	68	10.5	8.0	12.2	9.9	0.9
Nares‐orbit distance	18	20.6	19.1	22.2	21.0	0.8	68	22.8	19.1	25.2	21.6	1.2
Minimum interorbital distance	18	19.2	17.6	20.8	19.4	0.8	68	20.9	17.1	22.2	19.3	1.0
Basicaudal plate length	17	26.6	16.8	27.6	22.3	2.7	68	22.9	14.8	24.5	19.3	2.2

Abbreviation: SD, standard deviation.

**TABLE 5:** Frequency of meristic characters in *Loricaria catirina* sp. nov. (*n* = 18) and *L. teteae* sp. nov. (*n* = 77).

	Number of anterior lateral plates				
	16	17	18	19	20
*Loricaria catirina* sp. nov.		2	13^a^		2
*Loricaria teteae* sp. nov.	1	13	39^a^	21	3

	Number of coalesced lateral plates			
	14	15	16	17
*Loricaria catirina* sp. nov.	3	6	8^a^	
*Loricaria teteae* sp. nov.	15	36^a^	22	4

	Number of total lateral plates			
	32	33	34	35
*Loricaria catirina* sp. nov.	1	8	8^a^	
*Loricaria teteae* sp. nov.	5	41^a^	30	1

	Number of postanal plates			
	18	19	20	21
*Loricaria catirina* sp. nov.	1	12	4^a^	
*Loricaria teteae* sp. nov.	6	27	38^a^	6

^a^ The values of the holotype of each species.

urn:lsid:zoobank.org:act:ED216331‐095C‐4A9C‐9177‐F06EE90865F7.

*Loricaria* cf. *cataphracta*: Vieira et al., 2023: 21 (species inventory).

**Holotype**: MNRJ 56152, 181.3 mm SL, rio Munim, Povoado Cedro, Chapadinha Municipality, Maranhão State, Brazil, 03°50′19.25″S, 43°19′44.80″W, 25 March 2011, J. L. Nunes & Equipe LabAqua.

**Paratypes**: All from Munim River, Maranhão State, Brazil. CPUFMA 483, 2, 172.6–200.5 mm SL, Povoado Cedro, Chapadinha, 03°50′19.25″S, 43°19′44.80″W, 25 May 2011, Equipe LabAqua. CPUFMA 1607, 1, 188.0 mm SL, Povoado Cedro, Chapadinha, 03°50′19.25″S, 43°19′44.80″W, 20 January 2011, Equipe LabAqua. CICCAA 6920, 1, 176.3 mm SL, Presidente Juscelino, 02°55′39.2″S, 44°03′50.6″W, July 2022, M. Coelho, M. Paiva, R. Oliveira, L. Oliveira. CPUFMA 1608, 9, 153.7–195.0 mm SL, Povoado Cedro, Chapadinha, 03°50′19.25″S, 43°19′44.80″W, March 2011, Equipe LabAqua. UFRN 6238, 3, 153.7–195.0 mm SL, Povoado Cedro, Chapadinha, 03°50′19.25″S, 43°19′44.80″W, 25 March 2011, Equipe LabAqua. CPUFMA 1495, 1, 195.4 mm SL, Povoado Cedro, Chapadinha, 03°50′19.25″S, 43°19′44.80″W, 16 July 2010, Equipe LabAqua.

##### Diagnosis

*Loricaria catirina* is distinguished from its congeners, except *L. coximensis*, *L. holmbergi*, *L. lundbergi*, *L. nickeriensis*, *L. parnahybae*, *L. pumila*, *L. spinulifera,* and *L. turi*, by presence of polygonal, clearly separated plates in central abdominal region, interspersed by areas of naked skin and pectoral girdle covered by large, widely spaced plates (Figure 4a,c,d) (vs. abdominal region and pectoral girdle entirely covered by small, tightly packed plates without gaps; Figure 4b). *Loricaria catirina* differs from *L. lundbergi* by having greater basicaudal plate length (16.8%–27.6% vs. 9.96%–12.0% of HL); from *L. spinulifera* by having fewer oral papillae (14–16 vs. 20) and shallow, rounded postorbital notch (Figure 5b) (vs. well‐developed, angular postorbital notch); from *L. coximensis* and *L. nickeriensis* by reaching larger body size (over 150 mm SL vs. 100 mm and 130 mm SL, respectively); from *L. pumila* by having higher number of lateral plates (33–34 vs. 31–32) and poorly developed odontodes on predorsal plates (vs. well‐developed odontodes in predorsal region); from *L. holmbergi* by presence of shallow, rounded postorbital notch (Figure 5b) (vs. well‐developed, angular postorbital notch); *Loricaria catirina* differs from *L. parnahybae* by having basicaudal plate length greater than interorbital width (vs. plate equal in size to interorbital width), and by higher number of oral papillae (14–16 vs. 8–12); from *L. turi* by smaller minimum orbital diameter (14.9–20.2% vs. 30.1–43.1% of snout length) and greater inter‐nostril distance (17.1–20.8% vs. 10.16–16.6% of snout length).

##### Description

Morphometric and meristic data are presented in Tables 5 and 6. Body elongated, slender, dorsoventrally depressed. Caudal peduncle long, depressed. Greatest body width at cleithrum (11.5%–13.8% of SL). Lateral profile of head from snout tip to parieto‐supraoccipital slightly convex; from parieto‐supraoccipital to dorsal‐fin origin straight to slightly convex. Head triangular in dorsal view; lateral margins near opercular opening straight to slightly convex; snout acute, tip rounded. Lateral body profile from dorsal‐fin origin to end of caudal peduncle straight. Maximum body depth at origin of dorsal‐fin origin (8.0%–10.0% of SL). Postorbital notch present, rounded, shallow (Figure 5b). Maximum orbital diameter 20.4%–22.8% of HL.

Body covered by dermal plates, except ventral surface of head anterior to gill opening, portion of pectoral girdle, pelvic‐fin base and V‐shaped area surrounding anus. Dorsal body plates from snout tip to dorsal‐fin origin with weakly to moderately developed odontode ridges. Odontodes poorly developed along inner orbital margin. Supraoccipital posterior portion with two closely set parallel ridges, extending onto predorsal plates, diverging posteriorly. Nuchal plate with single median ridge.

Upper lip with numerous elongate fringes covering premaxillary region. Lower lip with numerous long filaments; marginal barbels simple. Maxillary barbel longer than marginal barbels of lower lip. Premaxillary teeth bicuspid, 2–4 per ramus (mode 3); tooth base elongate, slender; apex bicuspid. Tooth cusps conical to rounded; inner cusp larger, broader than outer cusp. Buccal papillae posterior to premaxillary teeth, 14–16 (mode 14, rarely 16), arranged in three rows: innermost papillae larger than premaxillary teeth; outer papillae similar in size or slightly larger. Dentary teeth 5–9 per ramus (mode 7 left, 6 right), similar to premaxillary teeth, but smaller, approximately half length of premaxillary teeth.

Total lateral plates 32–34 (mode 34, rarely 32). Anterior lateral plates 17–20 (mode 18), with two parallel odontode keels. Posterior lateral plates 14–16 (mode 16). Post‐anal plates 18–20 (mode 19). Abdominal lateral plates 8–11, rectangular, elongate. Pectoral girdle partially covered by small, widely spaced plates (Figure 4a). Median abdominal region with polygonal plates, plates larger in pre‐anal shield; anterior abdominal region with plates concentrated along midline, irregularly arranged, often spaced, surrounded by naked skin.

Dorsal fin, when depressed, reaching 9–10 lateral plates from origin. Dorsal spine longer than first branched ray; remaining rays progressively shorter. Pectoral fin, when depressed, reaching seven lateral plates from origin; pectoral spine and first branched ray equal in length; subsequent rays progressively shorter. Pelvic‐fin, when depressed, reaching 8–10 lateral plates. Anal‐fin spine reaching 7–8 lateral plates from lateral series; branched and unbranched rays equal in length.

##### Coloration in preserved specimens

Dorsal surface dark to light brown; ventral surface pale yellow to cream. Narrow darkened middorsal stripe from dorsal‐fin terminus to end of caudal peduncle. Head with irregular pattern of dark brown, pale yellow pigmentation from snout to posterior margin of orbits. Predorsal plates light brown; surrounding plates slightly lighter. Dorsal and pectoral fins pale yellow; distal margins darkened. Pelvic‐fin spine pale yellow; faint reddish‐orange pigmentation from mid‐length to spine tip; remaining rays, interradial membranes uniformly pale yellow. Anal fin with all rays pale yellow.

##### Sexual dimorphism

Among the individuals examined, 10 were identified as males and exhibited the same sexually dimorphic traits observed in other *Loricaria* species. Males possess thicker pectoral‐fin spine and modified teeth with smaller, rounded cusps, whereas females and juveniles have longer and more pointed cusps.

##### Distribution

*Loricaria catirina* is only known from the middle and lower course of the Munim River, Maranhão State, Brazil, in the Cerrado savannah area (Figure 6).

##### Conservation status

The extent of occurrence (EOO) of *L. catirina* in the lower Munim River was estimated at 149 km^2^, a value that, by itself, would meet the threshold for threatened categories under IUCN criteria B1 (IUCN, 2024), even considering the whole area of the basin (15,918 km^2^; NUGEO). However, geographic restriction alone does not necessarily imply that the species is under threat.

The Munim River basin lies in a portion of the Cerrado biome that forms part of the MATOPIBA agricultural expansion zone (an acronym for the states of Maranhão, Tocantins, Piauí, and Bahia), a region undergoing intense degradation of riparian forests, increasing agrochemical use, and growing anthropogenic pressure (Santos & Naval, 2022). Nevertheless, no information is currently available regarding population size, abundance, or tolerance to environmental change.

Given the lack of basic ecological and demographic data, it is premature to infer that regional threats are effectively impacting the species' viability. Therefore, based on IUCN (2024) guidelines, we propose that the species be classified as Data Deficient (DD). This category highlights the need for further research on distribution, ecology, habitat preferences, and population dynamics, which are essential for a more robust conservation assessment.

##### Etymology

The specific epithet ‘*catirina*’ honours Catirina, one of the main characters of the Bumba Meu Boi folkloric festival, a traditional cultural expression from the state of Maranhão, northeastern Brazil. Since 2019, the Bumba Meu Boi from Maranhão has been recognized by UNESCO as Intangible Cultural Heritage of Humanity.

#### 3.4.2 *Loricaria teteae* sp. nov.

**FIGURE 7:**
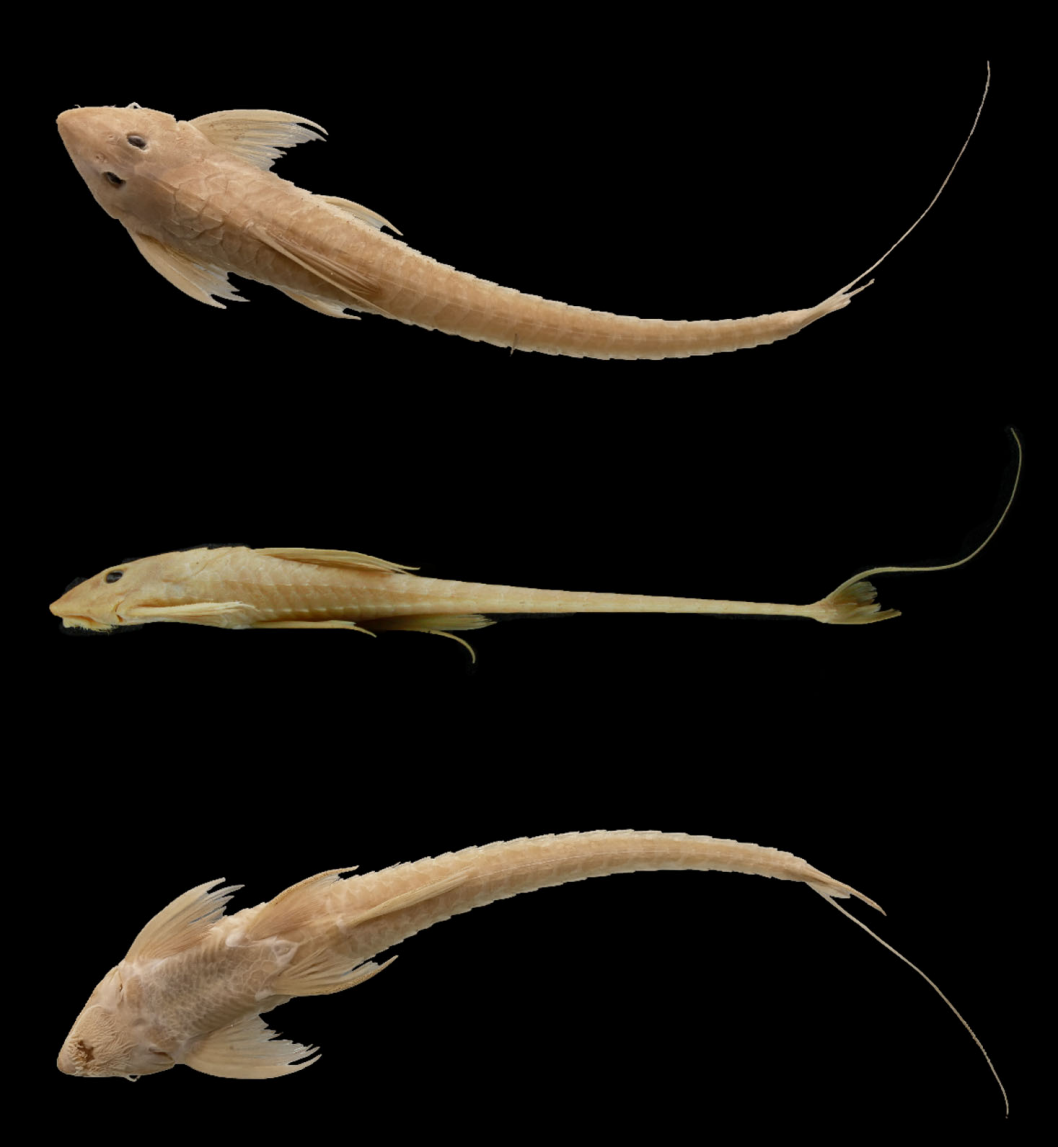
Holotype of *Loricaria teteae* MNRJ 56151, 188.1 mm standard length, male.

**FIGURE 8:**
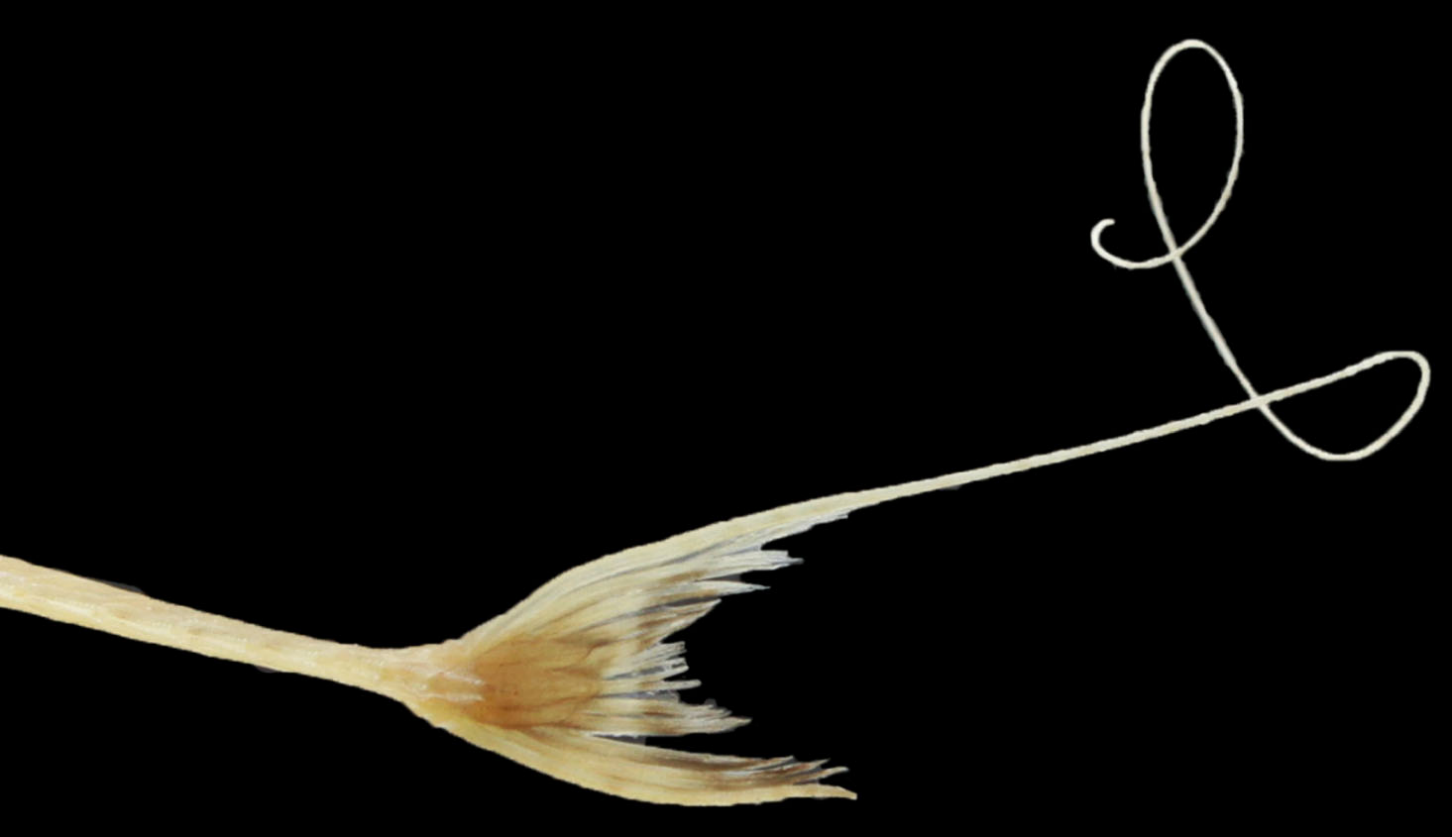
Caudal fin showing a conspicuous dark band along the distal portion of the rays. CPUFMA 1841.

**FIGURE 9:**
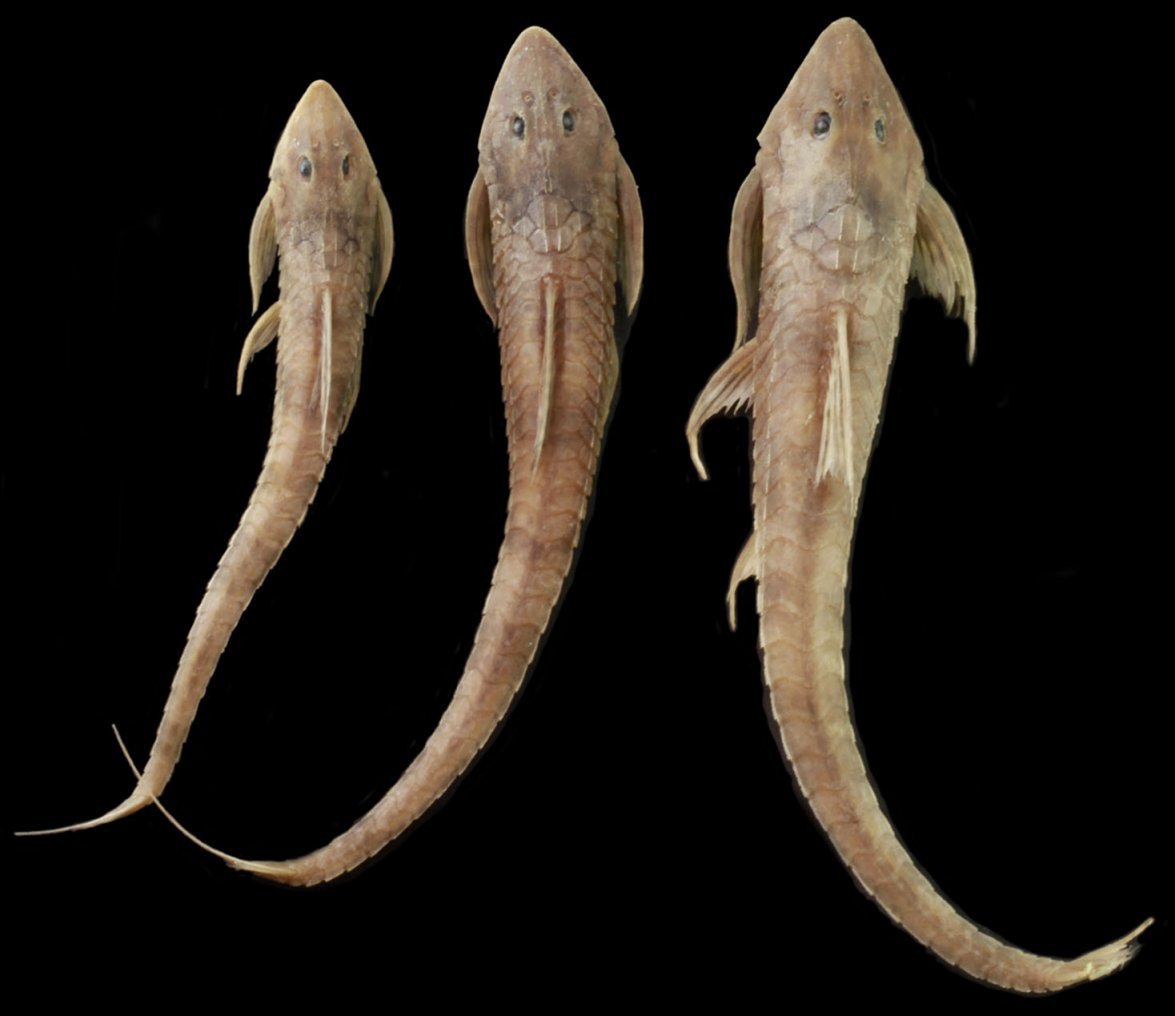
Variation in body coloration among individuals of *Loricaria teteae* sp. nov. Smaller individuals exhibit more prominent transverse bands along the body, whereas larger specimens tend to show more uniform coloration. From left to right: specimen measuring 176.9 mm SL, specimen with 189.1 mm SL, and specimen with 237.5 mm SL. All individuals from CPUFMA 4160.

Figures 4, 7, 5a, 8, 9, Tables 4 and 5.

urn:lsid:zoobank.org:act:16AFF78F‐AA79‐44BD‐879A‐174233646C3B.

*Loricaria* cf. *cataphracta*: Saraiva et al. (2021): 432 (comparative material list).

*Loricaria cataphracta*: Piorski et al. (1998): 18 (species list), Abreu et al. (2019) (species list in supplementary material).

**Holotype**: MNRJ 56151, 188.1 mm SL, rio Itapecuru, Povoado São Miguel, Rosário Municipality, Maranhão State, Brazil, 03°00′30.6″S, 44°16′00.1″W, 6 February 2013, N.M. Piorski.

**Paratypes**: All from Itapecuru River, Maranhão State, Brazil. CPUFMA 822, 5, 171.4–181.9 mm SL, Povoado São Miguel, Rosário, 3°00′30.6″S, 44°16′00.1″W, 06 February 2013, N. M. Piorski. CPUFMA 928, 3, 161.6–235.2 mm SL, Povoado Livre‐nos Deus, Colinas, 05°57′45.6″S, 44°12′58.1″W, 23 August 2009, N.M. Piorski. CPUFMA 650, 5, 144.6–196.1 mm SL, Povoado Santa Luzia, Rosário 03°02′38.3″S, 44°14′26.5″W, February 2012, N.M. Piorski. CPUFMA 313, 22, 125.9–249.8 mm SL, Rosário, October 2003, L.F. Costa. CPUFMA 314, 8, 189.4–241.3 mm SL, Rosário, outubro de 2003, L.F. Costa. CPUFMA 673, 2, 210.6–213.9 mm SL, Povoado Santa Luzia, Rosário, 03°02′38.3″S, 44°14′26.5″W, 21 August 2012, N.M. Piorski. CPUFMA 931, 9, 123.9–180.7 mm SL, Povoado Livre‐nos Deus, Colinas, 05°57′45.6″S, 44°12′58.1″W, 22 August 2009, N.M. Piorski. CPUFMA 1685, 1, 178.8 mm SL, Poço do Bento, Itapecuru‐Mirim, 15 September 2011, N.M. Piorski. CPUFMA 1817, 3, 213.6–238.4 mm SL, Povoado São Miguel, Rosário, 03°00′30.6″S, 44°16′00.1″W, December 2012, N.M. Piorski. CPUFMA 1818, 2, 150.7–177.4 mm SL, Povoado Santa Luzia, Rosário, 03°02′38.3″S, 44°14′26.5″W, 21 August 2012, N.M. Piorski & Equipe Labesp. CPUFMA 1820, 4, 188.2–229.6 mm SL, Povoado São Miguel, Rosário, 03°00′30.6″S, 44°16′00.1″W, December 2012, N.M. Piorski. CPUFMA 1841, 6, 181.7–240.9 mm SL, Povoado Santa Luzia, Rosário, 03°02′38.3″S, 44°14′26.5″W, 04 February 2013, N.M. Piorski & Equipe Labesp. CPUFMA 1868, 4, 183.9–247.1 mm SL, Rosário, 02°56′38.1″S, 44°14′42.0″W, July 2013, N.M. Piorski. CPUFMA 4160, 11, 188.2–229.6 mm SL, Povoado São Miguel, Rosário, 03°00′30.6″S, 44°16′00.1″W, January 2024, W. Guimarães.

##### Diagnosis

*Loricaria teteae* differs from all congeners, except *L. cataphracta*, *L. clavipinna*, *L. cuffyi*, *L. lata*, *L. nimairaco*, *L. piracicabae*, *L. thomasi* and *L. tucumanensis*, by having median abdominal region and pectoral girdle fully covered by dermal plates (Figure 4b) (vs. median abdominal region and pectoral girdle partially covered; Figure 4a,c,d). *Loricaria teteae* differs from *L. cataphracta* by having narrower head (12.1%–17.0% SL vs. 17.2%–25.0% SL) and smaller abdominal length (10.7%–13.8% vs. 14.3%–17.7% SL); from *L. clavipinna* by having greater interorbital distance (17.1%–22.5% HL vs. 16.7%–17.2% HL) and longer basicaudal plate (14.8%–24.5% HL vs. 11.9%–14.4% HL); from *L. cuffyi* by having conspicuous and deep postorbital notch (Figure 5a) (vs. notch inconspicuous, rounded and shallow); from *L. lata* by fewer anterior lateral plates (16–20 vs. 19–22) and more posterior plates (14–17, usually 15 vs. 12–14); from *L. nimairaco* by presence of dark spots on pectoral fin (vs. absence), and by absence of uniform black or dark‐brown pigmentation or paired longitudinal stripes extending from head to dorsal‐fin origin (vs. presence); from *L. piracicabae* by presence of bicuspid teeth and distinct postorbital notch (vs. unicuspid teeth and absence of postorbital notch); from *L. thomasi* by 14–16 buccal papillae posterior to premaxilla (vs. 25–29); from *L. tucumanensis* by larger minimum orbital diameter (12.6%–18.5% vs. 11.9%–12.1% of snout length), longer basicaudal plate (3.0%–4.0% SL vs. 2.4%–2.8% SL), and longer pectoral fin (57.8%–66% vs. 56.65%–57.9% of predorsal length). *Loricaria teteae* differs from geographically proximate species *L. turi* and *L. parnahybae* by presence of plates fully covering pectoral girdle (vs. pectoral girdle only partially covered). It also differs from *Loricaria catirina* by presence of deep, angular postorbital notch (Figure 5a) (vs. notch short, rounded).

##### Description

Morphometric and meristic data are presented in Tables 4 and 5. Body elongated, slender, dorsoventrally depressed. Caudal peduncle long, flattened. Maximum body width across cleithral region (10.9%–15.8% of SL). Lateral head profile from snout tip to parieto‐supraoccipital slightly convex; from parieto‐supraoccipital to dorsal‐fin origin straight to slightly convex. Head triangular in dorsal view; lateral margins near opercular opening straight to slightly convex. Lateral body profile from dorsal‐fin termination to caudal‐peduncle tip straight. Maximum body depth at dorsal‐fin origin (7.8%–11.1% SL). Postorbital notch well developed, angular (Figure 5a). Maximum orbital diameter 15.7%–23.1% of HL.

Body covered by dermal plates, except ventral head surface anterior to gill opening, pelvic‐fin base and V‐shaped area surrounding anus. Dorsal body plates from snout tip to dorsal‐fin origin with moderately developed odontodes. Posterior portion of supraoccipital with two closely set parallel odontode ridges, extending onto predorsal plates, becoming more widely spaced posteriorly. Nuchal plate with single median ridge.

Upper lip with numerous elongate fringes covering premaxillary region. Lower lip with numerous long filaments; marginal barbels simple. Maxillary barbel longer than marginal barbels of lower lip, occasionally reaching gill opening. Premaxillary teeth bicuspid, 2–4 per ramus (mode 3); tooth base elongate, slender; apex biscupid. Tooth cusps conical to rounded; inner cusp larger, broader than outer cusp. Buccal papillae posterior to premaxillary teeth 12–16 (mode 14, rarely 16), arranged in three rows; innermost papillae larger than premaxillary teeth; outer papillae similar in size or slightly larger. Dentary teeth 4–9 per ramus (mode 6 on both sides), similar to premaxillary teeth, but smaller, approximately half length.

Total lateral plates 32–35 (mode 33). Anterior lateral plates 16–20 (mode 18) with two parallel odontode keels. Posterior lateral plates 14–17 (mode 15). Post‐anal plates 18–21 (mode 20). Pectoral girdle fully covered by dermal plates. Abdominal lateral plates 7–11, rectangular, elongate. Median abdominal region with polygonal plates, larger on pre‐anal shield; median region entirely covered by small, irregularly arranged plates (Figure 4b).

Dorsal‐fin, when depressed, reaching 10–11 lateral plates from origin; dorsal‐fin spine longer than first branched ray; remaining rays progressively shorter posteriorly. Pectoral‐fin, when depressed, reaching 7–8 lateral plates from origin; pectoral spine, first branched ray equal in length; remaining rays shorter. Pelvic‐fin spine reaching 8–11 lateral plates. Anal‐fin spine reaching 7–9 lateral plates; branched, unbranched rays equal in length.

##### Coloration in preserved specimens

Body coloration light to dark brown; head slightly darker than trunk. Dorsal plates uniformly coloured, light brownish‐yellow. Dorsal fin with few scattered small spots. Pectoral and pelvic fins with several small spots. Anal fin hyaline. Caudal fin with dark band along distal portion of rays (Figure 8). Body with up to six faint transverse bands between dorsal‐fin origin and caudal peduncle; bands more distinct in smaller individuals (Figure 9).

**Sexual dimorphism**. As in *Loricaria catirina*.

##### Distribution

*Loricaria teteae* is known from the middle and lower sections of the Itapecuru River in Maranhão State, Brazil. This basin is located in the Cerrado biome and in the Amazon Estuary and Coastal Drainages ecoregion, as defined by Abell et al. (2008) (Figure 6).

##### Conservation status

The extent of occurrence (EOO) of *L. teteae* in the middle to lower Itapecuru river was estimated at 506 km^2^, which falls below the thresholds established by IUCN (2024) for threatened categories. However, no direct, ongoing, or immediate threat was identified to suggest the species as threatened.

The region encompassing known records is subject to anthropogenic pressures, particularly in the more urbanized portions of the basin, as noted by Leal et al. (2023), including riparian forest degradation and sewage pollution. Nevertheless, no data are currently available on population dynamics or the species' ability to persist in altered environments, preventing a robust assessment of extinction risk. Therefore, based on IUCN (2024) criteria, we propose that *Loricaria teteae* be categorized as Data Deficient (DD).

##### Etymology

The specific epithet ‘*teteae*’ honours Almerice da Silva Santos (1924–2011), better known as Dona Teté, an influential composer, singer, and dancer of Cacuriá from Maranhão. A central figure in Maranhão's popular culture, she played a pivotal role in the appreciation, preservation, and renewal of regional dance traditions, transmitting them across generations. Her cultural legacy was officially recognized in 2025, when the Government of Maranhão established the State Day of Cacuriá, celebrated annually on June 27, her birthday.

#### 3.4.3 Comparative material examined

***Loricaria apeltogaster***: MCP 13222, 2, 256.24–263.85 mm SL, Uruguay River, municipality of São Nicolau, Rio Grande do Sul State, Brazil. ANSP 193946, 1, 274,5 mm SL, Rio Uruguay, Rio Grande do Sul, Brazil.

***Loricaria birindellii***: ANSP 200819, 2, 200.3–274.5 mm SL, Rio Xingu, Pará, Brazil.

***Loricaria cataphracta***: ZMB 3160, lectotype, 1, 300.0 mm SL, South America. NRM 33, 1, 275.0 mm SL. ANSP 191020, 1, 151.3 mm SL, Rio Jari 10 km below Monte Dourado, Pará, Brazil, 1°13′30″S, 52°3′15″W. ANSP 180509, 1, 189.3 mm SL, Madre de Dios, Peru. ANSP 188757, 1, 183.5 mm SL, Rio Tapajós, Pará, Brazil. ANSP 191024, 121.5 mm SL, Rio Tocantins, Pará, Brazil. ANSP 8303, 1, 203.8 mm SL, Suriname.

***Loricaria clavipinna***: MCP 45735, 3, 174.3–196.1 mm SL, Quebrada Pinto Yaco, Purus River, Ucayali, Peru. ANSP 176144, 1, 150.8 mm SL, Rio Nanay, Peru. ANSP 176141, 1, 153.6 mm SL, Rio Nanay, Peru.

***Loricaria* cf. *holmbergi*:** ANSP 124122 Primavera. Alto Paraguay, Cacupe, Arroyo Ytyguazo.

***Loricaria lata***: CICCAA 00488, 1, 204.2 mm SL, Vermelho River, Tocantins River basin, municipality of Marabá, Pará State. CICCAA 00548, 1, 197.2 mm SL, Castanheira River, Tocantins River basin, municipality of Marabá, Pará state. CICCAA 00547, 2, 176.9–190.2 mm SL, Sororó River, Tocantins River basin, municipality of Marabá, Pará state. MCP 41985, 1, 159.16 mm SL, Córrego Roncador, triburary of Araguaia River, Tocantis River basin, municipality of Monte Carlos de Goiás, Goiás State. ANSP 187253, 1, 151.1 mm SL, Mato Grosso, Brazil.

***Loricaria lundbergi***: ANSP187412, paratype, 2, 84.2–92.4 mm SL, Rio Negro (Drenagem Amazonas), 13 km downriver of Carvoeiro, 38.9 km upriver of Moura 1°20′49″S 61°54′57″W.

***Loricaria parnahybae***: NWM 44854, lectotype, 1, 102.0 mm SL, Parnaíba River, municipality of Alto Parnaíba, Maranhão State. NWM 44823, paralectotype, 9, 66.2–109.3 mm SL, Parnaíba River, municipality of Alto Parnaíba, Maranhão State. NWM 74917, 7, 95.2–126.1 mm SL Parnaíba River, municipality of Alto Parnaíba, Maranhão State. MCP 23375, 2, 124.0–144.3 mm SL, Parnaíba River, Parnaíba River basin, municipality of Teresina, Piauí State. CPUFMA 2911, 2, 156.7–174.4 mm SL, Balsas River, Parnaíba River basin, Maranhão state. CPUFMA 932, 8, 131.6–163.2 mm SL, Parnaíba River, Parnaíba River basin, municipality of Coelho Neto, Maranhão State. CPUFMA 159, 4, 167.4–171.6 mm SL, Bolinha River, tributary of Balsas River, Parnaíba River basin, municipality of Balsas, Maranhão State. UFRN 1220, 1, 131.5 mm SL, Rio Sambito, Aroazes, Piauí, Brazil. UFRN 2717, 1, 140.1 mm SL, Rio Tají, Corrente, Piauí, Brazil. UFRN 2734, 1, 162.6 mm SL Rio Corrente, Corrente, Piauí, Brazil. UFRN 3080, 1, 145.7 mm SL, Rio Parnaíba, Alto do Parnaíba, Maranhão, Brazil; UFRN 3052, 1, 138.4 mm SL, Rio Lontras, Barreiras do Piauí, Piauí, Brazil. UFRN 1222, 4, 125.7–136.9 mm SL, Rio Sambito, Aroazes, Piauí, Brazil. UFRN 3761, 5, 130.5–149.3 mm SL, Rio Gurguéia, Corrente, Piauí, Brazil. UFRN 2731, 4, 116.6–130.0 mm SL Rio Corrente, Corrente, Piauí, Brazil. UFRN 2750, 2, 111.7–151.7 mm SL, Rio Corrente, Corrente, Piauí, Brazil; UFRN 3021, 1, 200.4 mm SL, Rio Uruçuí‐Vermelho, Barreiras do Piauí, Piauí, Brazil; UFRN 3757, 4, 130.7–134.6 mm SL, Rio Corrente, Corrente, Piauí, Brazil.

***Loricaria pumila***: ANSP 178689, paratype, 81.0 mm SL Rio Tocantins, 11.3 km downstream of Curucambaba, 24.8 km upstream from Maiuata. 2°2′22″S 49°17′26″W.

***Loricaria similima***: ANSP 178692, 2, 99.0–114.3 mm SL, Rio Amazonas, 39.1 km downstream of Gurupa, 27 km upstream of Serraria, Pará, Brazil, 1°13′58″S 51°20′50″W.

***Loricaria spinulifera***: ANSP 178691, paratype, 1, 78.6 mm SL, Rio Negro (Drenagem Amazonas) 10.6 km downriver of Leprosario, 14.8 km upriver of Manaus 3°6′0″S 60°9′33″W.

***Loricaria tucumanensis*** ANSP 83645, 2, 82.0–106.4 mm SL, Tarija, Bolivia.

**Loricaria turi: All from Turiaçu River basin, Maranhão, Brazil**. CPUFMA 3204, holotype, 192.2 mm SL, Lago do Rapa Cuia, 2°23′30.28″S 45°23′49.15″ W. CPUFMA 196, paratype, 4, 178.4–186.4 mm SL, Lago Mendes. CPUFMA 285, 1, 214.8 mm SL, Lago do Rapa Cuia. CPUFMA 221, 12, 159.2–203.3 mm SL, Lago Mendes, Turiaçu River basin. CPUFMA 345, 26, 164.6–186.9 mm SL, Lago do Quebra‐Pote. CPUFMA 351, 20, 157.6–200.4 mm SL, Lago do Arrodeador. CPUFMA 470, 10, 183.7–197.9 mm SL. CPUFMA 471, 14, 171.6–188.1 mm SL, Lago Mendes. CPUFMA 417, 15, 161.9–195.7 mm SL Lago do Quebra‐Pote.

## 4 DISCUSSION

The recognition of *Loricaria catirina* and *L. teteae* as distinct species is supported by the integration of diagnostic morphological characters and molecular species delimitation analyses. Integrative taxonomic approaches are particularly effective for species delimitation in morphologically conservative groups once they combine independent lines of evidence and reduce uncertainty in species boundaries (Dayrat, 2005; Padial et al., 2010). In Loricariinae, in which morphological differentiation is often subtle, the integration of complementary datasets provides a robust framework for species delimitation (de Almeida et al., 2024; Dopazo et al., 2025; Pinheiro et al., 2025).

Phylogenetic analyses placed *L. catirina* and *L. teteae* in a well‐supported clade together with *L. parnahybae*, indicating a close evolutionary relationship among lineages endemic from the Munim, Itapecuru, and Parnaíba river basins in Maranhão and Piauí states (Figure 2). In contrast, *L. turi* from Turiaçu River basin (Maranhão) was recovered in a sister clade, exhibiting a closer phylogenetic affinity with *L*. aff. *nickeriensis* from the Guiana Shield. This topology indicates that northeastern coastal drainages do not constitute a single evolutionary unit within *Loricaria* but instead harbour lineages with distinct biogeographic affinities, reflecting independent evolutionary histories, as previously suggested for freshwater fishes in the region (Abreu et al., 2019, 2020; Limeira‐Filho et al., 2024).

The placement of *L. turi* as sister to a lineage from French Guiana is similar to a pattern previously observed in other groups such as *Pseudoplatystoma* Bleeker, 1862, *Pimelodus* Lacepède, 1803, and *Leporinus* Agassiz, 1829. In these cases, species  from the Turiaçu River were found to be more closely related to Amazonian species than to those from northeastern drainages (Carvalho‐Costa et al., 2011; Limeira Filho et al., 2024; Nascimento et al., 2024). This pattern suggests a history marked by vicariant events among drainages in the region. In the case of the Turiaçu basin, factors such as the presence of paleo‐drainages and the uplift of the Serra do Tiracambu mountains may have played an important role in isolating the local ichthyofauna from that of nearby coastal basins (Abreu et al., 2019; Piorski, 2010; Soares Júnior et al., 2011).

Despite the geographic proximity of the Munim, Itapecuru, and Parnaíba river basins, phylogenetic structure and basin‐level endemism indicate that each system has functioned predominantly as an independent diversification unit. This geographic isolation likely promoted allopatric speciation, as evidenced by consistent morphological and genetic differences among the species. Several studies have demonstrated that speciation events in freshwater fishes are often linked to geological changes and long‐term hydrological isolation among river basins, which limit dispersal and gene flow and promote evolutionary divergence (Albert et al., 2020; Albert & Reis, 2011; Barreto et al., 2022; Cassemiro et al., 2023; Leroux et al., 2022).

Previous studies in northeastern Brazil suggested that Miocene–Pliocene sea‐level fluctuations promoted episodes of drainage rearrangement and intermittent hydrological connectivity among basins (Abreu et al., 2019, 2020; Cassemiro et al., 2023). However, the reciprocal monophyly of lineages from each basin and consistent morphological differentiation indicate that, despite potential past connections, these basins have remained effectively isolated in more recent evolutionary times. This scenario suggests that long‐term hydrological isolation outweighed transient connections, ultimately driving the allopatric diversification in *Loricaria* along coastal drainages.

Although the genetic difference between *L. catirina* and *L. parnahybae* is relatively low (2.2% for *cox1*), similar values have been reported between distinct species‐level lineages within *Loricaria*. Notably, Londoño‐Burbano et al. (2023) reported 2% divergence between *L. simillima* and *Loricaria* sp. ‘Paraguay’, both supported as distinct evolutionary lineages and not sister taxa in their topology. Similarly low divergences between species have been documented in other loricariids (e.g., Fagundes et al., 2020; Londoño‐Burbano & Britto, 2022; Lustosa‐Costa et al., 2024), indicating that the divergence values observed here fall within the expected range for the group.

Accurate species delimitation also has direct implications for interpreting historical distribution records. Vieira et al. (2023) reported *Loricaria* cf. *cataphracta* from the Munim River basin; however, *L. cataphracta* differs notably from *L. catirina* in abdominal plating and postorbital notch morphology. *Loricaria cataphracta* is widely distributed in South America, occurring in the Amazon basin and coastal rivers of the Guiana Shield (Thomas, 2011). In our phylogeny, *L. cataphracta* from different localities, such as the Tocantins, Amazon, and French Guiana rivers, formed a single cohesive lineage. This pattern was supported by most species delimitation methods, with a species‐level split supported only by GMYC. Based on our study, previous records of *Loricaria* cf. *cataphracta* from the Munim and Itapecuru basins (Saraiva et al., 2021; Vieira et al., 2023) correspond to the new species described herein. Thus, the distribution previously attributed to *L. cataphracta* in northeastern Brazil is revised, restricting its occurrence to the Amazon and Guana basins.

The species *Loricaria turi*, *L. parnahybae,* and *L. catirina* share the condition of an unplated pectoral girdle whereas *L. teteae* has this region completely covered by dermal plates, making it morphologically distinct from the other regional species. Thomas et al. (2013) and Londoño‐Burbano et al. (2023) suggested that a naked pectoral girdle may be a common trait among species distributed south of the Amazon basin, with a few exceptions. The identification of a species with a plated girdle in a northeastern coastal basin adds to these exceptions and suggests that this character may not reflect a clear biogeographic signal. Although the phylogenetic relationship found in this study does not indicate a direct Amazonian origin for these lineages, colonization patterns of coastal drainages from the Amazon have already been proposed for other fish groups (Hubert & Renno, 2006; Albert & Reis, 2011). Alternatively, it may represent cases of morphological convergence or retention of an ancestral trait in some lineages. Since not all species were included in the phylogeny presented here, broader phylogenetic and morphological studies of the genus are needed to test these hypotheses.

From a conservation perspective, both newly described species, *Loricaria catirina* and *L. teteae*, exhibit highly restricted extents of occurrence, falling below IUCN (2024) thresholds for threatened species. These species occur within the Cerrado savannah, recognized for its high biodiversity and under intense anthropogenic pressure in Brazil (Colli et al., 2020). In this region, agricultural expansion, particularly in the MATOPIBA zone (Maranhão, Tocantins, Piauí, and Bahia), has established a new agricultural frontier driven largely by soybean cultivation, leading to widespread deforestation and environmental degradation (Chamon et al., 2022; Polizel et al., 2021; Santos & Naval, 2022; Silva et al., 2023). Such changes affect not only terrestrial vegetation but also the associated aquatic environments, since the deforestation of riparian forests, increased erosion and intensified use of agrochemicals directly impact water quality and the integrity of riverine habitats (Silva et al., 2023). In this context, species with distributions restricted to a single coastal basin, such as those described herein, become particularly vulnerable to habitat loss and degradation.

In the Munim and Itapecuru river basins, where the new species occur, environmental impacts are evident and include riparian forest degradation, pollution, and intensive land use (Castro et al., 2018; dos Santos & Leal, 2013; Martins et al., 2022; Silva et al., 2023; Vieira et al., 2023). However, despite potential threats, no data are currently available on the precise geographic distribution, population size, relative abundance, demographic trends, or ecological tolerance of these new species. This data gap prevents a precise assessment of extinction risk. Therefore, based on IUCN (2024) criteria, we propose that both species be classified as ‘Data Deficient’, highlighting the urgent need for targeted studies on their ecology, population dynamics, and habitat use to support future conservation reassessments.

Overall, the descriptions of *L. catirina* and *L. teteae* reveal the presence of morphologically and genetically distinct species in northeastern Brazil's coastal drainages and support previous hypotheses that these basins function as important evolutionary units and areas of endemism (Hubert and Renno, 2006; Abreu et al., 2019). Our results are consistent with a diversification scenario driven by geographic isolation, likely shaped by vicariant events and the complex geological history of the region. By integrating morphological, molecular, and biogeographic evidence, this study contributes to refining species limits within *Loricaria* and clarifying patterns of diversification in northeastern coastal river systems, providing a robust taxonomic framework for future evolutionary and ecological studies.

## AUTHOR CONTRIBUTIONS

**Ananda. C. Serejo‐Saraiva**: Conceptualization; data curation; formal analysis; investigation; methodology; visualization; writing – original draft. **Nivaldo M. Piorski**: Conceptualization; investigation; methodology; data curation; writing – review and editing; photography; fieldwork. **Felipe P. Ottoni**: Conceptualization; resources; investigation; methodology; writing – review and editing; provided tissue samples and morphological material. **Mark H. Sabaj**: Conceptualization; methodology; resources; writing – review & editing; provided comparative material. **Sergio M. Q. Lima**: Conceptualization; methodology; formal analysis; data curation; writing – review and editing.

## FUNDING INFORMATION

This study was supported by a PhD scholarship to A.C.S.S. from Fundação de Amparo à Pesquisa e ao Desenvolvimento Científico e Tecnológico do Maranhão (FAPEMA, grant BD02692/21), a PhD Sandwich scholarship to A.C.S.S. from Coordenação de Aperfeiçoamento de Pessoal de Nível Superior (CAPES, grant 88,881.980664/2024‐01), and research grants from Conselho Nacional de Desenvolvimento Científico e Tecnológico (CNPq, grants 307974/2021‐9 and 306490/2024‐2 to FPO, and 306490/2024‐2 to SMQL).

## Supporting information


**TABLE S1.** Mitochondrial *cox1* sequences of *Loricaria* retrieved from public databases and used in the phylogenetic analyses.

## Data Availability

The data that support the findings of this study are available from the corresponding author upon reasonable request.
