# Supplementary material for: Integrative taxonomy reveals two new species of whiptail catfishes Loricaria (Siluriformes: Loricariidae) from northeastern Brazil
Source: J Fish Biol. 2026 Apr 8;108(6):2080–97. doi: 10.1111/jfb.70395 (PMC13357375; doi:10.1111/jfb.70395)
Supplement: Supplementary file 1 — TABLE S1. Mitochondrial cox1 sequences of Loricaria retrieved from public databases and used in the phylogenetic analyses. [file JFB-108-2080-s001.docx]

Supporting Information

Supplementary Material for:

Integrative taxonomy reveals two new species of whiptail catfishes, Loricaria (Siluriformes: Loricariidae), from northeastern Brazil

Journal of Fish Biology

Table S1. Mitochondrial COI sequences of *Loricaria* retrieved from public databases and used in the phylogenetic analyses.

| **Species** | **Voucher** | **Tissue number** | **Locality** | **Coordinates** | **Genbank** |
| --- | --- | --- | --- | --- | --- |
| *L. cataphracta* | N/A | GFSU12-209 | Maroni River, Saint-Laurent du Maroni, French Guiana, | 05°03'06.1"N 54°05'20.0"W | MZ051968.1 |
| *L. cataphracta* | N/A | GFSU12-208 | Maroni River, Saint-Laurent du Maroni, French Guiana, | 05°03'06.1"N 54°05'20.0"W | MZ050922.1 |
| *L. cataphracta* | N/A | GF06-470 | Maroni River, Saint-Laurent du Maroni, French Guiana, | 05°03'06.1"N 54°05'20.0"W | MZ052016.1 |
| *L. cataphracta* | N/A | GF06-470 | Maroni River, Saint-Laurent du Maroni, French Guiana, | 05°03'06.1"N 54°05'20.0"W | MZ051232.1 |
| *L. cataphracta* | INPA 43893 | INPA43893 | Nhamunda River, Amazonas, Brazil, | 01°41'27.2"S 57°25'20.3"W | KP772582.1 |
| *L.* cf. *cataphracta* | MCP 52212 | MCP52212 | Rio Amazonas, Santarém, Pará, Brazil | 02°14’07”S 54°48’13”W | OP407981.1 |
| *Loricaria* sp. | INPA060096 | TOC920 | Rio Tocantins, Cametá, Pará, Brazil | 02°15'32.4"S 49°30'03.6"W | OR733009.1 |
| *Loricaria* sp. | INPA060096 | TOC765 | Rio Tocantins, Cametá, Pará, Brazil | 02°15'32.4"S 49°30'03.6"W | OR733004.1 |
| *Loricaria* sp. | INPA060096 | TOC764 | Rio Tocantins, Cametá, Pará, Brazil | 02°15'32.4"S 49°30'03.6"W | OR733005.1 |
| *Loricaria* sp. | INPA060096 | TOC766 | Rio Tocantins, Cametá, Pará, Brazil | 02°15'32.4"S 49°30'03.6"W | OR733007.1 |
| *Loricaria* sp. | INPA060096 | TOC918 | Rio Tocantins, Cametá, Pará, Brazil | 02°15'32.4"S 49°30'03.6"W | OR733008.1 |
| *L.* aff *nickeriensis* | N/A | GFSU14-125 | Marouini River, St-Laurent-du-Maroni Saut Wayo, French Guiana | 02°40'36.8"N 53°59'57.5"W | MZ051100.1 |
| *L.* aff *nickeriensis* | N/A | GF00-120 | Marouini River, Antecume Pata, Maripasoula French Guiana, | 03°00'02.9"N 54°04'59.9"W | MZ051111.1 |
| *L.* aff *nickeriensis* | N/A | GF00-097 | Tampok River, Saut Pierkuru Maripasoula, French Guiana | 02°49'00.8"N 53°31'59.2"W | MZ051906.1 |
| *L.* aff *nickeriensis* | N/A | GF15-378 | Maroni River, Cayode, Tampok River, , French Guiana, | 03°23'24.4"N 53°55'33.6"W | MZ051588.1 |
| *L.* aff *nickeriensis* | N/A | GFSU14-324 | Marouini River, Langa Soula, French Guiana, | 02°51'31.0"N 53°58'38.3"W | MZ051209.1 |
| *L.* aff *nickeriensis* | N/A | GF00-098 | Tampok River, Saut Pierkuru Maripasoula, French Guiana | 02°49'00.8"N 53°31'59.2"W | MZ051265.1 |
| *Loricaria* sp. | INPA060006 | TOC759 | Rio Tocantins, Babaçulândia, Tocantins, Brazil | 07°05'06.0"S 47°36'46.8"W | OR733006.1 |
| *Loricaria* sp. | N/A | IIP656 | Peru | - | KT952449.1 |
| *L. simílima* | ROM uncataloged | ROM T-28093 | Pastaza River, Amazonas River Basin, Equador | - | OP407983.1 |
| *L. simillima* | MCP 46182 | MCP46182 | Jauaperi River, Caroebe, Roraima, Brazil | 00°54’47”N 59°34’23”W | OP407984.1 |
| *L. simillima* | MCP 46205 | MCP46205 | Jaburu Stream, Rorainópolis Roraima, Brazil | 00°37’15”N 60°31’05”W | OP407985.1 |
| *L. nimaraico* | ROM 107225 | T-24758 | Orteguaza River, Amazon Basin, Caquetá, Amazonas, Colômbia | 01°31’09”N 75°32’19”W | OP407977.1 |
| *L. nimaraico* | ROM 107265 | T-24827 | Orteguaza River, Amazon Basin, Caquetá, Amazonas, Colômbia | 01°39’29”N 75°32’31”W | OP407978.1 |
| *L.* cf. *cataphracta* | MCP 52233 | MCP52233 | Rio Amazonas, Santa Rita, Óbidos, Pará, Brazil | 02°02’34”S 55°24’34”W | OR407982.1 |
| *L.* cf*. cataphracta* | MCP 51629 | MCP 51629 | Rio Amazonas, Paraná Carareacá, Pará, Brazil | 02°10’55”S 54°52’45”W | OR407980.1 |
